# The Production and Purification of Therapeutic Antibodies: A Comprehensive Analysis of Process- and Product-Related Contaminants

**DOI:** 10.3390/biom16050738

**Published:** 2026-05-18

**Authors:** Kevin James, Andrej Kovac, Petra Majerova

**Affiliations:** 1 Institute of Neuroimmunology, Slovak Academy of Sciences, Dubravska Cesta 9, 84510 Bratislava, Slovakia; kevin.james@savba.sk (K.J.); andrej.kovac@savba.sk (A.K.); 2 Department of Galenic Pharmacy, Faculty of Pharmacy, Comenius University Bratislava, Odbojárov 65, 83104 Bratislava, Slovakia

**Keywords:** monoclonal antibodies, bispecific antibodies, host cell proteins, chromatography

## Abstract

The pharmaceutical industry has seen significant growth in the development of antibody-based therapeutics, especially monoclonal antibodies (mAbs) and bispecific antibodies (bsAbs), used in the treatment of cancer and neurodegenerative diseases. However, their production and purification remain challenging. It is difficult to achieve both high product yield and the strict purity required for clinical use. Downstream processing is expensive and often involves trade-offs between efficiency and product quality. In addition, current purification methods do not fully remove contaminants, especially host cell proteins, residual DNA, and protein aggregates, affecting the safety and effectiveness of the final product. Recent advances in purification technologies, such as improved chromatography techniques and alternative separation methods, have shown promise in addressing some of these limitations. Process optimization and the integration of continuous manufacturing approaches are being explored to enhance efficiency and scalability. Furthermore, increased regulatory expectations are driving the need for more robust and reproducible purification strategies. As the antibody therapeutics market continues to expand, optimizing manufacturing and purification processes is crucial to achieve cost efficiency and large-scale production. This article discusses the main challenges in antibody production and downstream purification, focusing on monoclonal and bispecific antibodies, and compares current strategies to increase yield, improve purity, and reduce contaminants.

## 1. Introduction

The production of antibodies and antibody-based therapeutics involves multiple downstream purification steps that are essential for achieving high purity and preserving structural integrity. A key challenge in these processes is the presence of both process- and product-related impurities. Process-related impurities originate from the expression system, most commonly animal cell cultures used for large-scale production. Despite their capability to yield substantial quantities of recombinant protein, these systems also contribute host cell proteins (HCPs) and other residual contaminants that must be efficiently eliminated during downstream processing, typically accompanied by some loss of the target product at each purification stage.

Conversely, product-related impurities stem from the physicochemical properties and inherent stability of the therapeutic protein. Attributes such as molecular complexity, elevated protein concentrations, and exposure to diverse mechanical and chemical stresses throughout manufacturing—including mixing, agitation, filtration, freeze–thaw cycles, and interfacial interactions—can facilitate the generation of aggregates, misfolded conformers, or other molecular variants that compromise product quality. These complexities are especially magnified in advanced molecular formats such as bispecific antibodies (bsAbs), necessitating rigorous control over both impurity clearance and product stability [1,2,3].

Given these considerations, optimizing the downstream purification processes is critical to effectively reduce both process- and product-related impurities while maintaining yield and structural integrity. Table 1 summarizes the key steps involved in downstream processing of antibody-based therapeutics, and Table 2 provides an overview of commonly used large-scale manufacturing equipment along with their advantages and limitations [4,5,6,7].

## 2. Product- and Process-Related Impurities

### 2.1. Product-Related Impurities

**Figure 1:**
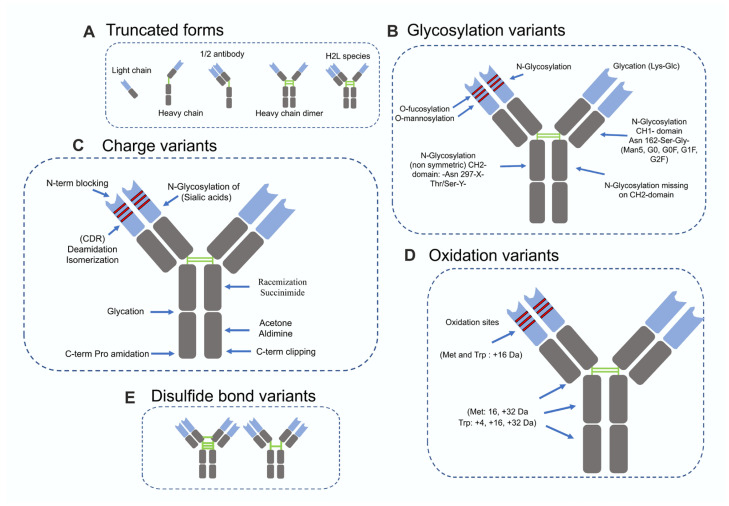
Product-related impurities in mAb production can be classified as follows: (**A**) fragments/truncated forms—low-molecular-weight species including half-antibodies, light- or heavy-chain fragments, or specific domain fragments (e.g., Fab or Fc). (**B**) Glycosylation variants—differences in glycan structures attached to the Fc region, including variations in monosaccharide composition, branching patterns, or the presence or absence of specific residues (e.g., fucose or sialic acid). (**C**) Charge variants—acidic or basic forms of the antibody that differ in net surface charge. (**D**) Oxidation variants—modifications resulting from the addition of oxygen atoms to susceptible amino acid residues, such as methionine, tryptophan, and histidine. (**E**) Disulfide bond variants—species with partially reduced disulfide bonds, incorrectly paired (scrambled) disulfide linkages, or unpaired cysteine residues (adapted from [22]).

**Figure 2:**
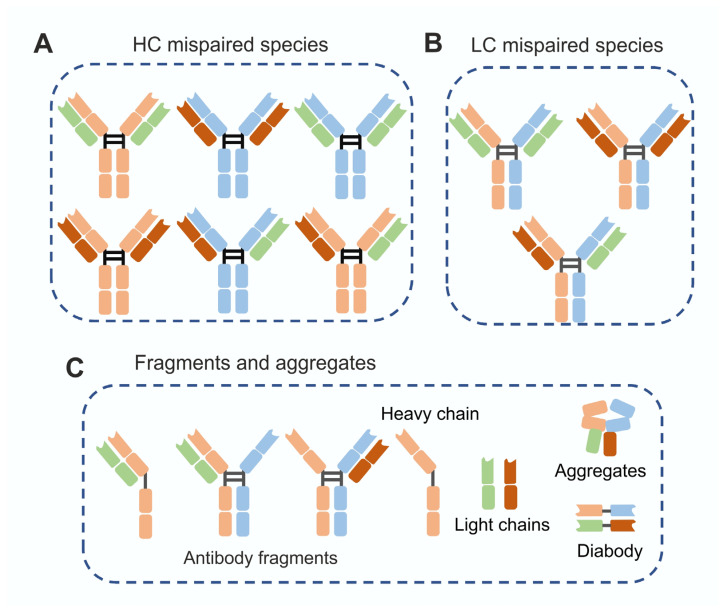
Common product-related impurities observed in bsAb production. (**A**,**B**) Symmetric mispaired species—these comprise mispaired heavy-chain (HC) and light-chain (LC) species generated through aberrant chain pairing during biosynthesis and assembly. (**C**) Orientation mismatches—species resulting from improper assembly or incomplete structures, including half-antibodies, three-quarter antibodies, isolated heavy- or light-chain species, diabodies, and nonspecific aggregates formed due to incomplete or misoriented chain assembly and aberrant association events (adapted from [2]).

Product-related impurities generate heterogeneous populations of the target protein encompassing variants that may exhibit either conserved or altered physicochemical properties. Variants with distinct structures, such as aggregates, are generally easier to separate, whereas impurities with similar physicochemical characteristics present a greater challenge. Figure 1 and Figure 2 illustrate common product-related impurities with both similar and dissimilar physicochemical properties observed during the production of monoclonal antibodies (mAbs) and bispecific antibodies (bsAbs).

Changes in pH, exposure to elevated temperatures or oxidative conditions, and variations in ionic strength can also affect the tertiary and quaternary structure, as well as the molecular orientation of the final product. In addition, host cell-related factors, including undesirable post-translational modifications (PTMs), proteolytic degradation, cell culture conditions, and genetic instability, have been shown to significantly impact mAb quality attributes [8].

Common impurities in mAb production include incorrect chain assembly products and truncation variants encompassing heavy chains (HC), light chains (LC), and single-chain variable fragments (scFvs), as well as post-translational modification (PTM) variants such as glycosylation, charge, oxidation, and disulfide bond variants (Figure 1). Truncated forms of mAbs arise from inefficient or aberrant assembly of heavy and light chains, resulting in species such as isolated light chains (LC), heavy chains (HC), half-antibodies, heavy-chain dimers, and H2L variants (Figure 1A). Glycosylation occurs in both Fc and Fab regions, with common types including N-glycosylation, O-fucosylation, and O-mannosylation, which can affect binding affinity, solubility, immunogenicity, stability, and protein folding (Figure 1B) [9]. Fc glycosylation typically involves N-glycosylation with core fucosylation at Asn297 in the CH2 domain and modulates effector functions such as ADCC [10], while variable-region glycosylation is less common but present in antibodies like cetuximab [11].

Charge variants include acidic forms (e.g., sialylation, deamidation, N-terminal modifications, and glycation) and basic forms (e.g., C-terminal lysine clipping and succinimide formation), as well as structural variants such as racemization and acetone aldimine formation (Figure 1C) [12]. These modifications can alter pI, binding affinity, and stability; for example, CDR deamidation can reduce binding up to 14-fold [13], while glycation can mask positive charges and affect stability [14]. C-terminal lysine clipping generally has minimal impact on potency [15].

Oxidation of methionine and tryptophan residues can disrupt hydrophobic interactions and induce conformational changes, increasing polarity and reducing antibody stability (Figure 1D) [16]. Disulfide bond variants, including mismatches or non-native formations among the 16 bonds in IgG antibodies, can compromise structural integrity and folding (Figure 1E) [17].

Due to the greater structural complexity of bsAbs compared with mAbs, product-related impurities tend to form at increased frequency and diversity during bsAb production [18]. Depending on the bsAb format (e.g., symmetric, asymmetric, or fragment-based) and the host cell line used, various mismatched by-product species may be generated (Figure 2). Although many of these mismatches can be removed through downstream purification strategies, some homodimers and mispaired structures exhibit physicochemical properties similar to those of the desired product and may therefore co-purify [19]. This co-purification contributes to reduced overall process yields commonly observed in bsAb manufacturing and represents a major challenge in their production [20]. Among the different bsAb formats, fragment-based constructs tend to generate the highest levels of product-related impurities due to their increased ability for aggregation [20,21].

### 2.2. Process-Related Impurities

Host cell proteins and contaminants (HCPs/HCC) are process-related impurities derived from the intracellular metabolic and biosynthetic machinery of the cells used to produce therapeutic recombinant proteins. Additional process-related impurities include residual host cell DNA, endotoxins, and viruses, all of which have been reported to compromise product quality and contribute to significant losses during downstream processing and manufacturing [23].

Residual host cell DNA and bacterial endotoxins primarily originate during production, whereas viral contaminants may arise from viral vectors, such as adeno-associated virus (AAV), used for protein expression. Regulatory authorities, including the WHO and FDA, have established strict guidelines to control these impurities. Residual DNA is limited to 10 ng per dose with fragment sizes below 200 base pairs [24], while bacterial endotoxins must not exceed a maximum pyrogenic dose of 5 EU/kg for parenteral drugs [25]. In addition, viral clearance is ensured through validated inactivation steps and nanofiltration using 30–35 nm filters to remove viruses such as adenoviruses, retroviruses, and herpes simplex viruses [26].

Although these measures effectively minimize most process-related impurities and ensure high safety standards for antibody-based therapies, HCPs remain particularly challenging to eliminate. Residual HCPs may persist due to strong interactions with recombinant proteins or co-elution driven by similar physicochemical properties [27]. Furthermore, nonspecific interactions can promote their association with target proteins throughout downstream purification, although many are reduced through binding to chromatography resins [28].

Importantly, HCPs can negatively affect product stability and safety. For example, proteolytic HCPs, such as cathepsins, may induce antibody degradation, while others can trigger adverse immunological responses with potentially severe clinical consequences [29]. Currently, there are no strict official guidelines or limits specifying permissible levels of HCPs in the therapeutic products by any regulatory bodies. However, as a standard, regulatory bodies advise a standard limit of 100 ppm [30]. Accordingly, the main focus of this review is to provide an overview of recent advances in the removal of process-related impurities, with particular emphasis on HCP clearance strategies.

Table 3 summarizes several HCPs commonly detected during antibody production, along with their potential impacts on antibody quality and immunogenicity.

However, these guidelines do not specify the individual HCP species. Strategies to reduce HCP levels include optimizing upstream conditions, such as adding media supplements like folic acid, thiamine, riboflavin, ascorbic acid, and insulin and essential amino acids and vitamins, or adjusting culture parameters, such as mild hypothermic conditions, which have been shown to decrease HCP levels by up to 36% [30]. Optimized cell culture duration, timing of harvest, and cell viability are critical. Additionally, minimizing cellular stress—by maintaining optimal pH, temperature, and osmolality, reducing shear stress from agitation, and media replacement—can further lower HCP levels. Finally, using knockout or knockdown cell lines for problematic HCPs, as discussed further, provides another effective strategy.

## 3. Expression Systems for the Production of Therapeutic Proteins

Mammalian cell lines serve as the major backbone in the industry for production of antibodies, due to their lower immunogenic risk, which is attributed to phenotypic and genetic similarities, as well as their ability to perform essential PTMs. Mammalian expression systems include Chinese hamster ovary (CHO) cells, NS0 murine myeloma cells, human embryonic retinal cells (PER.C6^®^), and Human Embryonic Kidney (HEK) 293 cells [40]. Protein production is a complex multistage process. Therefore, selecting an appropriate cell line and employing a compatible expression system are essential for achieving optimal protein expression. However, a key limitation of using cell culture-based expression systems is the potential contamination of the final product with HCPs, which, even at trace levels, can significantly impact antibody quality, function, and overall yields [42].

### 3.1. Chinese Hamster Ovary (CHO) Cells

The CHO cell line is the most widely used for therapeutic protein production, recognized for its high production efficiency, robust tolerance to manufacturing stresses, ability to perform human-like PTMs, and low risk of triggering cross-species immune responses. Over 70% of all recombinant immunotherapies are produced in CHO cells [43,44]. While mAb production in CHO cells can be scaled up to 10 g/L, bsAbs often exhibit lower expression yields ranging from 1 to 3 g/L [42].

CHO cells naturally lack dihydrofolate reductase (DHFR) activity. By introducing a DHFR gene on the expression vector, only cells that take up the vector can survive in a medium lacking DHFR or in the presence of methotrexate (MTX). This allows the selective growth of cells that are producing the therapeutic protein and serves as a selection and gene amplification system [45].

Examples of mAbs produced in CHO cells include Rituximab, Bevacizumab, Panitumumab, and Tocilizumab [46]. Blinatumomab is an innovative bsAb used for the treatment of specific forms of acute lymphoblastic leukemia (ALL). It is the second bsAb to receive FDA approval and shows enhanced antigen-binding affinity when manufactured in CHO cells compared with production in *Escherichia coli* [47].

Proteome and glycoproteome analyses have identified over 6000 HCPs across various CHO cell lines [48]. A detailed evaluation of HCPs in 29 FDA-approved and commercially available mAbs and bsAbs produced in CHO cells revealed 79 common HCPs present in most therapeutics within the standard limit of 100 ppm, as determined by LC-MS profiling. However, certain HCP species, including protein S20, flavin reductase, phospholipase B-like protein, and protein S100, were detected above the limit [35]. Several studies have reported a high presence of clusterin, lipoprotein lipase (LPL), a common HCP arising from the degradation of polysorbates (e.g., polysorbate 80 and 20), which are frequently employed to prevent aggregation and degradation of therapeutic proteins [49]. Since LPL is particularly challenging to remove during downstream processing [50], Chiu et al. generated an LPL-knockout CHO cell line using the CRISPR system, which led to a significant reduction of up to 57% [37]. Similarly, matriptase-1 knockout CHO-K1 cells were generated using transcription activator-like effector nucleases (TALENs) to eliminate the serine protease matriptase-1 (MT-SP1), which is primarily responsible for proteolytic degradation of recombinant antibodies. These knockout cells exhibited comparable viability and higher antibody yields compared to wild-type CHO cells [51].

Unlike these examples, which targeted a single gene, Kol et al. performed multiplex genome editing to target up to 14 different HCP genes [52]. Testing production of the monoclonal antibody Rituximab showed that 6 x KO cells produced more antibody than 11 x KO cells and slightly more than wild-type CHO cells, whereas 14 x KO cells had very low productivity. Downstream purification using a three-step process further demonstrated minimal HCP levels in the knockout cells, with the 11 x KO cells showing the lowest HCP load (3 ppm), followed by 6 x KO cells (18 ppm), compared to wild-type cells (45 ppm).

Although generating knockout cell lines involves considerable production time and cost, these studies collectively demonstrate that targeted gene knockouts can reduce specific HCP loads during production and enhance expression of the gene of interest. This, in turn, can simplify downstream purification and reduce associated costs.

### 3.2. Human Embryonic Kidney (HEK) Cells

HEK cells are derived from embryonic kidney tissue and widely used for transient and stable expression of recombinant proteins. HEK cells are expected to become a major platform for novel antibody-based therapeutics due to their human-like PTMs and physiologically relevant glycosylation patterns [53]. Several HEK cell line variants are widely employed depending on expression requirements, including Expi293, HEK 293E (expressing the Epstein–Barr virus nuclear antigen), HEK 293F (Freestyle), HEK 293G, HEK 293T (expressing the SV40 T antigen), HEK 293S (suspension culture), and HEK 293SG lacking N-glycans due to knockout of the N-acetyl-glucosaminyltransferase I (GnT-I) gene. HEK-based systems are cost-effective and can produce large amounts of proteins efficiently [54], while Expi293 cells can produce up to six times more protein than standard HEK 293 cells.

Nevertheless, the potential of HEK cells should be further explored, particularly for producing antibody fragments that are difficult to express in other systems. This capability could be highly beneficial for the production of bsAbs and trispecific antibodies [55]. Despite these advantages, CHO cells remain the predominant choice for both research and industrial production because HEK cells require additional biosafety measures to prevent contamination from human viruses.

### 3.3. Human Embryonic Retinal Cells

Other human-derived production systems include human embryonic retinal cells, such as PER.C6^®^, which are obtained by immortalizing retinal cells from human embryos using the E1 genes of adenovirus 5 DNA. These cells exhibit minimal immunogenicity, do not add potentially harmful and reactive glycans to recombinant proteins, can rapidly grow to densities of 5 × 10^6^ cells/mL, and have stable IgG expression [56].

### 3.4. Cell-Free Expression

Cell-free expression is an alternative method for producing proteins without relying on living cells. This approach utilizes cellular extracts containing only the essential components required for transcription and translation, thereby facilitating protein production without the cells. Various lysates have been developed, including *E. coli* lysate, wheat germ extract (WGE), insect cell extract, yeast extract, and eukaryotic extracts such as rabbit reticulocytes, CHO, and HeLa (immortal human epithelial cells) lysates. Xu et al. employed an *E. coli*-based cell-free system (Xpress CF) to design four knob-into-hole (KiH) antibody targeting tumor antigen epithelial cell adhesion molecule (EpCAM) and human epidermal growth factor receptor 2 (HER2) [57].

While cell-free expression offers significant advantages in terms of production time and the potential for minimal HCP contamination, several challenges remain for large-scale applications. Scalable production is a major concern, as production costs and maintaining extract activity are comparatively high. Additional limitations include the risk of endotoxin contamination when using *E. coli*-based extracts [58]. Regulatory approval is another major limitation [59]. A summary of expression systems is provided in Table 4.

## 4. Monitoring of HCPs

Monitoring HCPs in each cell culture batch is challenging, as many HCPs are small and may bind to the therapeutic protein. While enzyme-linked immunosorbent assay (ELISA) is considered the analytical gold standard for evaluating HCP presence [76], one- and two-dimensional liquid chromatography–mass spectrometry (LC–MS) provides powerful platforms for comprehensive HCP profiling [77]. Commercial ELISA kits are widely used due to their simplicity, cost-effectiveness, and compatibility with routine monitoring [78]. However, ELISA has limitations: it may fail to detect novel or unspecified HCPs and cannot easily identify individual HCP species. SDS-PAGE can provide quantitative assessment but is rarely used because of low specificity and limited sensitivity for low-abundance HCPs [79].

LC-MS provides high-resolution profiling of HCPs. Data-dependent acquisition (DDA) enables identification of novel HCPs, although method optimization can be challenging. For precise, sensitive, and quantitative detection, targeted MS techniques—such as multiple reaction monitoring (MRM) and parallel reaction monitoring (PRM)—can detect HCPs approximately 10 ppm. With proper optimization, MS-based methods offer reproducible and highly accurate quantitative HCP analysis [80]. Independent studies indicate that combining immuno-based assays with mass spectrometry provides a more comprehensive analysis of HCPs in the final product. This approach enables improved detection of HCPs, including nonspecific tightly bound species, making it a more effective method for HCP identification [80]. It was demonstrated that capillary electrophoresis combined with LC offered broader HCP coverage [81].

Recent advances in HCP detection include Orbitrap Astral mass spectrometry combined with high-field asymmetric waveform ion mobility spectrometry (FAIMS), enabling the detection of HCPs at concentrations below 10 ng/mg and the identification of up to 236 unique HCPs [82]. Despite these developments, many HCPs remain unidentified, highlighting the critical need for continued discovery of novel HCP species and further improvement of analytical methods to ensure the safety of antibody-based therapeutics. In this context, regulatory bodies should broaden their approach by incorporating orthogonal techniques such as LC–MS [83]. Furthermore, current detection strategies can be enhanced through the use of more specific HCP detection kits, tagging approaches for improved identification, and molecular methods to eliminate problematic HCPs. Detection kits should also be adaptable to newly identified HCPs, compatible with different production cell lines, and sensitive enough to detect low-abundance proteins. Purification of bsAbs is more challenging than that of standard monoclonal antibodies due to their unique and complex structures. The capture stage of protein purification primarily relies on chromatographic techniques, which are essential for separating structural by-products and HCPs. Several studies have shown that customizing conventional chromatographic approaches used for mAbs can improve yields and enhance separation from impurities such as HCPs and low- or high-molecular-weight (HMW) species in bsAb production [84].

Non-IgG-like antibody formats, such as BiTEs, DARTs, and tandem diabodies (TandAbs), are particularly prone to aggregation, which further complicates their purification [85]. Key factors influencing purification strategy include antibody heterodimerization format, selection of chromatographic resins, surface charge, hydrodynamic radius, hydrophobicity, glycosylation, and other structural attributes.

### 4.1. Affinity Chromatography for Antibody Purification

Currently, Protein A-based affinity chromatography is the most widely used single-step method for antibody purification. Protein A has strong affinity for the Fc region as well as the VH domain of antibodies, making it effective for conventional antibody purification [86]. IgG typically binds to five domains of Protein A—E, D, A, B, and C4—in a pH-dependent manner. The B domain, which exhibits the highest binding affinity, was used as the basis to engineer Z-domain resins.

Improved Z-domain resins can bind both the Fc and VH3 regions of antibodies at neutral pH and remain bound under alkaline conditions, with antibody elution typically achieved using acidic or low-pH buffers [87]. This multi-binding ability is both advantageous and challenging for modified heterodimeric antibodies, such as bsAbs and trispecific antibodies. Careful design of VH domains is critical, as the VH3 subfamily can bind non-covalently to Protein A resins, potentially complicating the separation of by-products formed from heterodimeric antibodies [88].

Protein A chromatography is particularly useful for separating half-antibodies containing only one Fc and Fab arm, which generally have lower binding affinity compared to fully structured mAbs or bsAbs. Modern Protein A resins, such as MabSelect SuRe LX, MabSelect SuRe, and MabSelect PrismA, have shown strong performance in separating various by-products generated during bsAb production. MabSelect PrismA, in particular, can differentiate bsAb by-products lacking one Fab arm, one light chain, or a knob half-antibody, due to its additional binding affinity to the VH3 region, unlike older resins that only bind CH2–CH3 and may fail to capture bsAbs lacking a light chain [89,90].

Recently, a cellulose-based resin, Fibro PrismA, containing the same Protein A ligand as MabSelect PrismA, has shown promising results. Zhang et al. demonstrated that Fibro PrismA provided superior resolution in separating difficult single-arm species from asymmetric IgG-like bsAbs, achieving yields up to 80.2% with shorter retention times under similar pH gradient elution conditions [91]. Supporting these findings, Napoleone et al. scaled up production of a low-titer tetravalent bsAb using piggyBac transposon-mediated HEK-293 cells. Using Fibro PrismA at high flow rates (30 mL/min), they achieved high-purity recovery within 4.5 h, significantly reducing processing time compared to conventional Protein A columns of similar size [92].

Protein A chromatography, despite its widespread use, has notable drawbacks, including low binding capacity, strong acidic elution conditions, ligand leaching, short shelf life, and high cost [93]. Nevertheless, its simplicity makes it the preferred choice for initial capture steps to remove process-related impurities.

Other affinity proteins, such as Protein G and Protein L, can also be used. Protein G, like Protein A, binds the heavy chain; however, its stability and binding affinity are generally lower than Protein A. Protein L, derived from *Peptostreptococcus magnus*, specifically binds the variable region of κ light chains, making it suitable for capturing bsAbs lacking a Fc fragment [94]. Because Protein L binds in the Fab region, by-products such as half-antibodies or three-quarter antibodies, which contain only one light chain, exhibit lower binding and can be separated. This property allows Protein L-based chromatography to further separate functional versus non-functional Fab arms, enabling purification of monovalent and bivalent bsAbs [85].

Downstream purification using Protein L can be enhanced with a three-step system involving Protein A, KappaSelect, and LambdaFabSelect resins. This approach is particularly useful for bsAbs with common heavy chains and heterodimeric light chains. Potential mispairings, such as κκ or λλ chains, are first captured by Protein A resins, and the desired bsAbs with two distinct light chains (κλ) are subsequently captured using KappaSelect and LambdaFabSelect resins [95].

Limitations of these resins include variability in binding affinity depending on the bsAb structure—for example, bsAbs with only one κ light chain bind differently than those with two κ chains—and the high cost of the resins must also be considered [96]. The combination of multiple chromatographic modalities can further enhance overall product purity, as certain process-related impurities have binding affinities similar to the target bsAb, making such combination strategies widely employed in both the capture and polishing stages of bsAb purification.

### 4.2. Size-Exclusion Chromatography (SEC)

Size-exclusion chromatography is one of the most widely used chromatographic techniques for the analytical characterization of biomolecules based on their size and shape [97]. For bsAbs, SEC offers a key advantage in enabling the detection and monitoring of aggregates and mispaired fragments [98].

Recent advances in ultra-high-performance size-exclusion chromatography (UHP-SEC) have led to substantial improvements in analytical performance, including higher resolution and reduced analysis times. UHP-SEC employs shorter columns with ≈3 µm particles, enabling operation at pressures up to 250–480 bar [97]. A recent study demonstrated that ultra-high-performance size-exclusion chromatography (UHP-SEC) can effectively differentiate FDA-approved therapeutic monoclonal antibodies based on variations in molecular size and hydrodynamic and conformational properties [99]. Optimization of the mobile phase composition according to antibody characteristics improves separation efficiency and reduces analysis time; phosphate-buffered saline (PBS) and ammonium acetate are widely employed under neutral/alkaline and acidic conditions, respectively [100].

To further enhance separation performance, mixed-mode SEC has been introduced, in which additional interactions—such as hydrophobicity, hydrogen bonding, and electrostatic effects—are exploited alongside molecular weight and hydrodynamic radius. This approach is particularly valuable for resolving bsAbs with similar sizes but differing physicochemical properties [100]. Because of the minimal size differences between bsAbs and their parental antibodies, Yang and team reported that high-efficiency SEC columns with 3 µm particle sizes (e.g., Zenix SEC 300 or Agilent Bio SEC-300) provided effective purification of half-molecule exchange IgG4 bsAbs, with improved resolution obtained through mixed-mode interactions [101]. Similarly, high-resolution SEC using a 300 × 4.6 mm column with 1.7 µm particle size and 200 Å pore diameter enabled the differentiation of scFv–IgG bsAb size variants generated as by-products of cysteine residue incorporation for enhanced stability [102].

Furthermore, the integration of mixed-mode SEC with mass spectrometry enables precise mass discrimination between desired bsAb constructs and undesired homodimeric species, providing an additional level of structural characterization [103].

### 4.3. Hydrophobic Interaction Chromatography (HIC)

Hydrophobic interaction chromatography separates proteins based on differences in their surface hydrophobicity.

High-salt conditions promote interactions between exposed hydrophobic residues on the protein surface and hydrophobic ligands on the stationary phase, while subsequent salt reduction weakens these interactions and enables protein elution [19].

HIC is widely used to distinguish mispaired homodimers from correctly assembled antibody species, making it an effective tool in monoclonal and bispecific antibody production workflows [1]. However, excessively high salt concentrations can result in irreversible binding of mAbs and bsAbs to the resin, leading to reduced recovery yields [104,105]. Consequently, parameters such as salt type and concentration, column dimensions, flow rate, and operating temperature must be carefully optimized to maximize separation efficiency and protein recovery [106].

HIC was employed to separate structural variants of trastuzumab and demonstrated that modifications to standard protocols—such as using longer columns and sodium chloride as the mobile phase—resulted in enhanced resolution, reduced elution times, and improved variant separation [107]. Additional strategies to facilitate protein elution include the incorporation of mobile-phase additives such as 5% hexylene glycol or 1 M arginine, which weaken hydrophobic interactions between proteins and the stationary phase [108].

Notably, extended interaction with the resin at elevated temperatures has been shown to induce conformational changes in certain bsAbs, resulting in multiple elution peaks and reduced chromatographic resolution [109].

### 4.4. Ion Exchange Chromatography (IEX)

Ion exchange chromatography is an effective method for downstream purification of therapeutic mAbs and bsAbs, provided the charge properties and isoelectric point (pI) of the molecules are known. IEX can effectively reduce impurities such as host cell proteins, mispaired bsAb variants, DNA, and homodimer aggregates, thereby facilitating subsequent downstream processing [110].

Cation exchange chromatography (CEX) relies on charge-based separation, wherein proteins with net positive charge in the mobile phase interact non-covalently with negatively charged ion-exchange resins. Proteins bind to cation exchange resins with varying strengths and are subsequently eluted using buffers with increasing ionic strength or pH, typically ranging from 5.0 to 8.1. At lower pH, positively charged antibodies bind strongly to the resin, and elution is commonly achieved using sodium chloride. For bsAbs, this approach is particularly effective for separating homodimers from correctly assembled bsAb formats with differing isoelectric points (pI) [111]. Loading buffer pH, usually adjusted to 1–4 units away from the bsAb’s pI, and the salt gradient are key factors in sequential elution during CEX. Salt gradients have been shown to provide effective separation for traditional monoclonal antibodies; however, for complex bispecific antibodies, this approach often produces multiple elution peaks. To achieve higher resolution for bsAbs with closely similar pI values—such as species with mispaired light chains or homodimers—pH gradients have proven more effective, allowing differentiation of bsAb structures with pI differences as small as 0.1 units. This was further demonstrated in a study in which a common light chain IgG-like bsAb was successfully separated from its homodimer, with pI values of 8.95 and 8.36, respectively, using a MonoS 10/100 GL column and a pH gradient from 6.5 to 8.0 [112]. Alternatively, alkaline pH conditions near the pI of the target bsAb have been employed to purify bsAbs from monoclonal Ab–diabody aggregates containing mispaired disulfide bonds. Using POROS^®^ HS50 cation exchange columns and SP Sepharose HP for polishing, this approach achieved aggregate reductions of up to 99.3%, with potential for further optimization at large scale [113].

Both reversible and irreversible conformational changes, resulting in multiple elution peaks of monoclonal antibodies, have been reported by several studies. These changes are largely independent of pH, resin type, ligand chemistry, or aggregate presence, and are instead influenced by prolonged interaction times of mAbs and bsAbs with the column at varying binding strengths [114,115].

Another challenge is that the use of strong chromatographic conditions or elevated temperatures can lead to protein denaturation. This can be mitigated by preserving antibody structure through glycosylation, employing alkaline pH buffers, and adding stabilizing agents such as arginine to the loading buffer, which helps reduce both low- and high-molecular-weight aggregates and other impurities [116]. To further enhance purification efficiency, Duivelshof et al. compared different bsAbs and mAbs and found that pH gradients were more effective than salt gradients. The addition of solvents such as isopropyl alcohol (IPA), ethanol (EtOH), and acetonitrile (ACN) in the mobile phase markedly improved variant separation, though it also increased elution times. Optimization of the mobile-phase solvent composition can further enhance resolution, with the optimal solvent combination potentially differing for each bsAb construct [117].

Anion exchange chromatography (AEX) utilizes columns packed with positively charged resins, making it suitable for monoclonal antibodies and bispecific antibodies that exhibit neutral to positive charges. Under these conditions, the antibodies typically do not bind to the resin and are collected in the flow-through, while impurities are retained and subsequently eluted using salt gradients [118].

To enhance purity, especially in the presence of multiple impurity types, weak partitioning chromatography (WPC) is frequently employed. Unlike AEX, which is operated under conditions minimizing product-resin interactions and allowing impurities to bind to the stationary phase, WPC uses an isocratic method with an optimized mobile phase that promotes partial binding of the product. The elution buffer pH, usually ranging from 7 to 11, facilitates the release of partially bound bsAbs, enabling improved separation of closely related species [119]. While AEX is commonly used during initial capture steps, WPC is more effective in polishing stages.

Similar to CEX, AEX and WPC can be combined to remove impurities such as HCPs, host cell DNA, and protein aggregates. Liang et al. demonstrated that using a single-step POROS 50 HQ column in AEX mode efficiently cleared HCPs and DNA, and the subsequent application of WPC in the polishing step significantly increased the purity of knob-into-hole (KiH) bsAbs [110]. A similar strategy using Capto adhere (mixed-mode) anion exchange chromatography for KiH bsAbs achieved a 60% recovery rate and 98% purity from cell culture fluid, while also providing considerable viral clearance. Optimal loading conditions of pH 7.8  ±  0.2 and conductivity below 3.0 milliSiemens/centimeter (mS/cm) contributed to high yields [120].

Multiple studies have highlighted the potential of AEX in combination with other chromatographic techniques as part of two- or multi-step purification systems for various bsAb formats, including WuXiBodies with relatively low pI values compared to mAbs, and fragment-based Bispecific killer engager (BiKE) and trispecific killer engagers (TriKE) [111,120,121].

### 4.5. Mixed-Mode Chromatography

Neutral proteins are often difficult to separate and may require process optimization or buffer adjustment to enhance binding. Additional challenges in bsAb purification include aggregation—particularly in constructs containing scFvs—and the presence of HCPs in the product. BsAbs with scFvs are especially prone to aggregation, and conventional pH-gradient elution methods frequently fail to achieve high yields of purified protein [88,122]. Aggregation often occurs within chromatography columns, complicating elution and reducing recovery, which underscores the importance of optimizing column selection and loading concentrations based on the specific bsAb format for efficient purification [123].

For years, researchers have explored single-step chromatography to save time and reduce downstream costs. However, this approach may compromise the removal of product-related impurities. Given the structural complexity of bsAbs, mixed-mode chromatography (MMC), which combines multiple separation mechanisms in a single resin, offers significant advantages. Numerous studies have demonstrated the effectiveness of commercially available multimodal resins—such as Capto MMC, Capto adhere, Diamond MMC (Cytiva), TOYOPEARL MX-Trp 650M (Tosoh Bioscience) as well as other platforms like hydrophobic charge induction chromatography (HCIC) and ceramic hydroxyapatite, for the purification of diverse bsAb formats [19,124].

**Figure 3:**
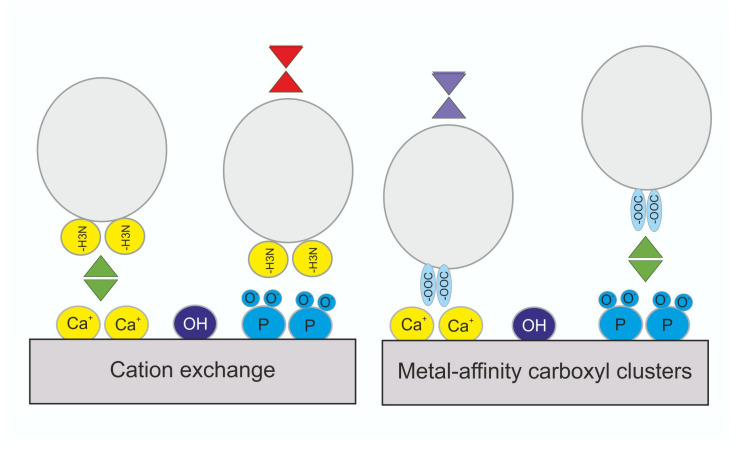
Ceramic hydroxyapatite chromatography. This technique combines cation exchange and metal affinity interaction mechanisms. The crystalline calcium phosphate matrix interacts with proteins through two distinct modes: cation exchange and calcium-mediated metal affinity. Elution is achieved using a NaCl gradient to release molecules bound via cation exchange, while a phosphate gradient is used to elute molecules bound through calcium-mediated interactions (metal affinity) [129].

Ceramic hydroxyapatite (CHT) chromatography is based on a hydroxyapatite matrix (Ca_10_(PO_4_)_6_(OH)_2_) containing five positively charged calcium pairs (C-sites) and phosphate triplets (P-sites). Its dual-mode binding combines metal affinity via the calcium sites, which interact with negatively charged HCPs, and cation exchange via the phosphate sites, which bind positively charged proteins (Figure 3). Typically, carboxyl groups on proteins interact with calcium sites (C-sites), whereas amino groups interact with phosphate sites (P-sites). Elution is achieved by increasing salt or phosphate concentrations, often via gradient methods, as NaCl can weaken repulsive interactions. Due to its ability to remove both product- and process-related impurities and to separate species with similar physical properties, CHT is widely applied for purifying biological molecules—including antibodies, antibody fragments, and viruses—during both initial capture and polishing steps [125].

Compared to CEX, CHT has demonstrated superior control of high-molecular-weight (HMW) and low-molecular-weight (LMW) impurities, achieving a reduction in HMW species to approximately 0.5% and a 48% decrease in LMW species, indicating its suitability for both symmetric and asymmetric bsAb formats.

Additionally, CHT efficiently reduces HCPs, including polysorbate-80-associated degradative enzymes, lipases, carboxypeptidase, and 78 kDa glucose-regulated proteins, yielding highly pure bsAb heterodimers [126].

CHT also provides effective separation of bsAb-related impurities such as Fc homodimers, half-antibody fragments, persistent low-molecular-weight species, and closely resembling bsAb formats reported in the study [127]. In the same study, two elution strategies were compared: CHT with a sodium phosphate linear gradient (CHT-P) and CHT with a sodium chloride gradient (CHT-NaCl). CHT-P provided superior separation of HMW and LMW aggregates in larger bsAbs but was less effective at HCP removal, whereas CHT-NaCl efficiently eluted bsAbs similar in size to IgG but with limited LMW clearance. Both methods achieved an average purity of 97%.

A 200 L pilot-scale study further optimized CHT by using arginine hydrochloride as the eluent, in addition to conventional phosphate- or sodium chloride-based eluents, to reduce hydrophobic interactions between highly hydrophobic bsAbs and the resin. This approach improved both purity (99%) and yield (80.7%) compared to Capto MMC and Capto adhere chromatography [128].

Commercial multimodal resins such as Capto MMC combine electrostatic (cation exchange), hydrophobic, and thiophilic interactions to enable selective protein separation. These resins are salt-tolerant and feature a rigid agarose matrix, allowing high flow rates. N-benzoyl-homocysteine ligands mediate the multiple interaction modes, and protein elution depends on pH for ionic interactions and salt gradients for hydrophobic interactions.

Capto MMC has demonstrated significant efficacy in removing aggregates from scFv-containing bsAbs. Chen et al. observed a 17–35-fold reduction in HCPs after Protein A capture followed by Capto Butyl ImpRes and Capto adhere MMC polishing, with improved loading densities and impurity removal in flow-through steps. Aggregate content decreased from 20% post-Protein A chromatography to 0.7% with linear gradient elution and 2.6% with stepwise elution [123].

Comparative studies between Capto MMC and Diamond MMC Mustang (Bestchrom) for asymmetric bsAb purification reported purities of 96.5% and 97.4%, respectively, from 69.6% after Protein A capture, with average yields of 63.2% and 62.1% [130]. For complex asymmetric bsAb formats such as WuXiBodies, Capto MMC effectively separated homodimers, aggregates, light chain mispaired by-products, and half-antibodies compared with IEX [131]. Aggregate removal efficiency with Capto MMC can vary between 1 and 5%, depending on the bsAb structure and hydrophobicity. Two studies by the same group reported aggregate reduction from 11.1% to 1.2% using dual pH–NaCl gradient elution [132,133].

The presence of a weakly acidic carboxyl functional group in Capto MMC resins renders the net resin charge highly dependent on buffer pH. As a result, electrostatic interactions may be insufficient to counterbalance hydrophobic interactions, which can hinder efficient elution of the target protein. In contrast, Capto adhere ImpRes (Cytiva) is a hydrophobic anion-exchange resin functionalized with N-benzyl-N-methyl ethanolamine ligands, conferring strong anion-exchange properties and enhanced electrostatic interaction capacity. This enables effective elution using salt gradients, even for negatively charged antibodies at pH values above their isoelectric point (pI) [19].

An optimization study evaluating Capto adhere in both bind-and-elute and flow-through polishing modes for monoclonal and bispecific antibodies demonstrated a 6% reduction in HMW species, along with a substantial decrease in HCP levels. The resin further exhibited efficient HCP clearance and the capacity to achieve above-average yields when process parameters were tailored to specific bsAb formats [124].

Capto adhere has been successfully applied for the purification and polishing of bsAbs under conditions of pH 7.90 ± 0.1 and conductivity of approximately 5.0 mS/cm, resulting in product purities exceeding 98% and average yields of ~60% [120]. Notably, for bsAbs with high surface hydrophobicity, Capto adhere demonstrated superior performance in removing by-products during polishing compared to the cation-exchange-based Capto MMC.

Furthermore, a two-column purification strategy incorporating Capto adhere achieved final impurity levels as low as 0.5% HMW and 0.4% LMW species, with an overall yield of 50.5% using Protein L-based MabSelect VL. This performance surpasses that of comparable three-column workflows incorporating Capto MMC, which yielded 5.9% HMW and 1.8% LMW species, with a slightly lower overall yield of 48.3% [134].

Another example of chromatography combining weak cation-exchange and hydrophobic interaction mechanisms is Toyopearl MX-Trp-650M. This resin incorporates a tryptophan ligand, in which the carboxyl group mediates weak cation-exchange interactions, while the indole ring enables hydrophobic interactions. It has been successfully applied to the purification of κλ bispecific antibodies (bsAbs) from IgG homodimers, mispaired species, and aggregates using a sequential NaCl gradient. Elution at approximately 75 mM NaCl was sufficient to recover the κλ bsAb, whereas homodimeric impurities were primarily detected in the flow-through or required higher salt concentrations (up to 500 mM NaCl) for elution [131]. However, several studies indicate that high purity and yield using this approach are achievable only for specific bsAb formats, which limits its broader applicability [19].

For bsAbs with more diverse and complex architectures, resins with tailored multimodal properties, such as Eshmuno CMX, have demonstrated improved purification performance and higher yields [131]. Eshmuno CMX combines cation-exchange functionality with moderate hydrophobic interaction, enabling effective separation of structurally complex molecules, including bsAbs and trispecific antibodies. The resin is functionalized with 2-amino-4-methylpentanoic acid ligands, which provide balanced hydrophobic interactions and facilitate efficient elution compared to more strongly hydrophobic multimodal resins such as Capto MMC.

For antibodies exhibiting a broad range of hydrophobicity, the partition coefficient (Kp) has been utilized as a high-throughput screening (HTS) parameter to identify optimal ligand chemistries and separation conditions. Koehnlein et al. evaluated multiple resins, including Eshmuno CMX, Capto MMC ImpRes, and Nuvia cPrime, and demonstrated that 2-amino-4-methylpentanoic acid provides moderate hydrophobic interaction strength relative to simpler functional groups. This balanced interaction profile helps mitigate excessive hydrophobic binding while maintaining effective separation. Using this approach, bsAb purification achieved approximately 70% yield and 97% purity [131].

Similarly, Song et al. evaluated the purification of hydrophobic asymmetric bsAbs that closely resemble homodimeric species, comparing Eshmuno CPX with other multimodal cation-exchange resins such as Capto MMC, Toyopearl MX-Trp-650M, and Nuvia cPrime.

Through systematic HTS optimization using pH and salt gradients, Eshmuno CPX demonstrated superior performance, achieving yields of 77%, reducing HCP levels from 5643 ppm to 7.5 ppm, and reaching purities exceeding 99%. Enhanced binding capacity was also observed under optimized conditions [128].

Despite these advances, the growing diversity of multimodal chromatography (MMC) platforms makes the selection of optimal resins and process conditions increasingly complex. Even with HTS-based optimization, trace levels of both process- and product-related impurities may persist after advanced purification steps. Therefore, a comprehensive set of parameters must be considered, including protein concentration, molecular size, net charge, isoelectric point, binding site preferences, partition coefficient (Kp), buffer composition, resin chemistry, interaction mechanisms, retention behavior, and overall process yield.

The Kp, which reflects the relative binding affinity of the target protein compared to co-existing impurities, serves as a powerful HTS metric for defining optimal separation conditions. Kp values are influenced by variables such as pH, conductivity, and resin type, and can be used to determine the separation factor under different operating conditions. A higher separation factor generally correlates with improved resolution of by-products [135]. Chen et al. reported that a minimum separation factor of 10 is required to achieve effective separation between the desired product and impurities. In their study, the highest separation factor (19) was obtained using the mixed-mode Eshmuno HCX resin [136].

In addition, logarithmic Kp values have been shown to be particularly useful for distinguishing half-antibody fragments from intact bsAbs and serve as reliable predictors of binding behavior for antibody fragments and aggregates, complementing MMC-based purification strategies [137]. Table 5 summarizes the major purification techniques, highlighting their advantages, limitations, and suitability for different bsAb formats.

## 5. Alternative Approaches for Polishing Techniques for Large-Scale Production

### 5.1. Conventional Batch Chromatography

Conventional batch chromatography typically employs a single Protein A column and is therefore time-intensive and operationally inefficient. In addition, the full binding capacity of the resin is often not utilized, resulting in increased buffer consumption per purification cycle and consequently higher operational costs [159]. Another major limitation of batch chromatography is the trade-off between purity and yield arising from overlapping adsorption regions of impurities and the target product, which can lead to substantial product losses. Although this effect can be mitigated by adjusting the column load, such modifications typically decrease overall process productivity [160]. Despite its limitations, batch chromatography remains widely used for laboratory-scale production of therapeutic proteins due to its procedural simplicity and ease of implementation.

### 5.2. Continuous Chromatography

In contrast, from both cost efficiency and productivity perspectives, continuous chromatography represents a compelling alternative for the manufacture of recombinant fusion proteins. Continuous chromatography employs a cyclic, multicolumn configuration—such as simulated moving bed (SMB) or periodic counter-current chromatography (PCC)—that enables uninterrupted feed loading and product collection. Multiple columns operate in parallel while sequentially performing loading, washing, elution, cleaning, and re-equilibration steps. This integrated, continuous operation significantly improves resin utilization, reduces buffer consumption and equipment footprint, and lowers operational costs, while achieving yields and product purity comparable to those obtained with conventional batch chromatography. Consequently, continuous chromatography is well-suited for large-scale biopharmaceutical manufacturing [159].

### 5.3. Multicolumn Chromatography (MCC)

MCC is a widely adopted form of continuous chromatography that employs multiple small columns operating in parallel in a defined sequence to maximize interaction between the target molecule and the stationary phase. Typically, three to four columns operate simultaneously, with each column assigned to a specific step of the purification cycle. In MCC, the concept of breakthrough capture refers to the retention of target molecules that bypass the initial column due to saturation and are subsequently captured by downstream columns. During cyclic operation, once a column becomes saturated, the feed is redirected to the next column, while the saturated column undergoes washing, elution, and regeneration steps [161].

Several MCC configurations have been developed to optimize monoclonal antibody purification. For example, Angarita et al. employed a dual-flow, twin-column counter-current chromatography system (Capture SMB), which achieved a higher yield (89%) compared with batch chromatography using Amsphere™ and MabSelect Sure resins [162]. One variation in MCC is SMB chromatography, which is designed to approximate true counter-current operation by enabling the stationary and mobile phases to move in opposite directions. This is accomplished through precise and periodic valve switching that simulates a moving bed, thereby maximizing resin–sample interactions. Similar to other MCC systems, SMB employs interconnected columns operating in a continuous mode.

The effectiveness of SMB has been demonstrated using commercial platforms such as the Cadence BioSMB PD system, which achieved a 75% mAb recovery from a 25 L harvested cell culture, along with a 4.5 log or more than 99.99% reduction in HCPs and a 50% reduction in aggregate content [163]. In SMB processes, the outlet streams are typically divided into an extract fraction, containing strongly binding target molecules, and a raffinate fraction, containing weakly binding species. This approach has proven effective for purifying scFvs. For instance, Cristancho and Seidel-Morgenstern reported purification of scFvs from cell culture supernatant using an open-loop, three-zone pH-gradient SMB process, achieving a 91% yield—9% higher than batch chromatography—along with an 11-fold increase in productivity and reduced buffer consumption [164]. Despite these advantages, SMB technology has limitations when applied to complex mixtures containing multiple target proteins. Due to its restricted ability to process multiple product fractions simultaneously, SMB is not well suited for the purification of two or more products, thereby limiting its industrial application for therapeutic protein purification.

### 5.4. Multicolumn Counter Current Solvent Gradient Purification (MCSGP)

MCSGP, in contrast to SMB chromatography, enables three-fraction separation using buffer gradients while employing conventional stationary phases and columns [165]. Typically implemented as a two- or three-column system with automated valve switching, MCSGP has emerged as a robust purification and polishing technique for monoclonal antibodies, bispecific antibodies, and antibody fragments. Müller-Späth et al. reported efficient monoclonal antibody isolation from cell culture supernatant using Protein A, multimodal anion exchange, and cation exchange resins in an MCSGP process, achieving 89–91% yields, ~97% purity, and improved HCP clearance relative to batch chromatography [166].

Further studies by the same group evaluated the applicability of MCSGP to commercially available mAbs, including Avastin^®^, Herceptin^®^, and Erbitux^®^, exhibiting charge heterogeneity. These investigations showed enhanced purity, improved yields, and preserved bioactivity relative to optimized batch processes [165]. Similarly, twin-column MCSGP has been applied to the purification of common light-chain bsAbs, achieving near-complete removal of closely related homodimeric impurities with yields up to 87%—approximately 50% higher than batch chromatography—along with aggregate levels of ~1%, DNA content below 10%, and HCP levels below 30 ppm in the final product [166]. Collectively, these studies highlight MCSGP as a highly effective alternative for polishing steps, offering enhanced resolution and process accuracy.

### 5.5. Periodic Counter-Current Chromatography (PCCC)

PCCC represents another continuous counter-current purification strategy. A typical PCCC system consists of two to four columns connected in series, with column functions periodically switched to maximize interactions between the stationary and mobile phases. Column number and switching times are optimized according to breakthrough behavior and desorption kinetics [167]. Following sample loading, column saturation is detected—commonly via UV absorbance—triggering column role switching and initiation of washing, elution, and regeneration steps. PCCC has been particularly effective in capture operations, where its continuous nature enables high resin utilization and consistent product recovery.

Comparative studies indicate that PCCC achieves purification performance, resin lifetime (up to 250 cycles), and HCP clearance comparable to batch chromatography, while offering advantages in reduced equipment footprint and capital costs [168]. With appropriate process optimization, PCCC has demonstrated the potential to deliver higher yields and product purity, supporting its suitability for industrial-scale antibody manufacturing [160]. Notably, Tustian et al. reported high-capacity capture of bsAbs using MabSelect SuRe Protein A resin in a PCCC configuration, achieving product purities of 98.1–98.5%, HCP levels of 210–229 ppm, and yields of 83–92.4% [169]. Nevertheless, PCCC commonly depends on UV-triggered switching and empirically defined step durations, which, together with limited use of advanced process analytics, can limit process optimization and flexibility [168].

### 5.6. Tangential Flow Filtration (TFF)

Tangential flow filtration is a versatile size-based separation technique in which the feed flows tangentially across the membrane, allowing selective separation of target proteins from impurities. In this configuration, the parallel flow minimizes membrane fouling by preventing accumulation of impurities, while permitting the passage of smaller contaminants and retaining the target proteins (retentate). TFF effectively concentrates proteins and significantly reduces HCP content. Important operational parameters—such as transmembrane pressure (TMP), crossflow velocity, and tangential flow rate—play critical roles in separation efficiency, reducing membrane fouling and enhancing membrane reusability and operational lifetime, which are common limitations in direct flow filtration [170,171].

Despite its advantages, conventional TFF has certain limitations, including low recovery rates, extended processing times due to recirculation of the retentate, and potential structural alterations in proteins caused by high shear forces during pumping. To address these issues, single-pass tangential flow filtration (SP-TFF) has been developed. Unlike traditional TFF, SP-TFF achieves protein separation in a single pass, eliminating recirculation and minimizing unnecessary interaction of the target protein with the membrane [172].

Using SP-TFF, highly concentrated and purified mAb solutions can be obtained, achieving low impurity levels (DNA < 2 ppm, HCP < 29 ppm) and yields above 96% [173].

SP-TFF performance depends on flow rate and membrane area; lower flow rates and longer residence times can improve protein concentration but may induce aggregation, while high-concentration feeds may promote membrane fouling and reduce product recovery efficiency [174].

### 5.7. High-Performance Tangential Flow Filtration (HP-TFF)

HP-TFF is an advanced filtration technique that offers enhanced selectivity by controlling the transmembrane pressure gradient, enabling the purification of biomolecules with similar sizes and charges. HP-TFF membranes are typically positively charged, allowing positively charged antibodies to be retained as the retentate, while negatively charged contaminants pass through into the permeate. Cellulose-based membranes with a 100 kDa molecular weight cut-off (MWCO) have demonstrated up to 10-fold improved removal of HCPs, achieving 98% recovery of Fab’2 antibody fragments at an industrial scale—a 12% improvement over conventional methods [175]. HP-TFF has also been effective in clearing aggregates, particularly for bsAbs; for example, a 300 kDa cut-off membrane with a 50 cm^2^ area successfully removed aggregates from scFv-IgG bsAbs produced in CHO cells [176].

Achieving high purity with HP-TFF requires large membrane areas, which increases buffer consumption and processing time. Although the membranes are reusable with long lifespans, repeated use can result in fouling. Additionally, maintaining uniform flow distribution and consistent transmembrane pressure becomes more challenging at large scales [19,177]. Despite these limitations, HP-TFF represents a powerful single-stage purification approach with significant potential for high-yield, high-purity protein production.

### 5.8. Charged Ultrafiltration (UF) Membranes

An emerging alternative to conventional chromatographic methods in polishing steps is the use of charged ultrafiltration membranes. In this approach, the membrane surface charge is tailored to the target protein, enabling selective retention or passage and thereby isolating the protein of interest from residual impurities [178]. These membranes are typically polymeric and can have their surface charge modified according to process requirements. Commonly used materials include polyether sulfone (PES), polysulfide (PSU), polyvinylidene fluoride (PVDF), and polypropylene (PP). One of the earliest quantitative studies on charged UF membranes was conducted by Mehta et al., who demonstrated how membrane and protein charge influence protein passage through the pores using charged cellulose membranes [179]. Charged UF membranes have also been shown to separate proteins with similar isoelectric points but different sizes. For example, Arunkumar and Etzel (2021) successfully separated α-lactalbumin from β-lactoglobulin using a 300 kDa UF membrane, achieving 87% purity of the isolated ALA [180]. While charged UF membranes offer scalability and efficiency for protein purification, they can impose higher interfacial stresses on antibodies due to charge-based interactions, which may impact protein stability compared with traditional chromatographic techniques [180].

Several studies have highlighted that although chromatographic techniques generally offer high yields, protein denaturation and aggregation can still occur during or after purification. Factors contributing to these effects include interactions between proteins and resins, protein concentration, the type of resin and ligands used, as well as low pH and temperature conditions during elution. Such destabilization often manifests as double or multiple peaks in chromatograms, underscoring the need to carefully manage protein–resin interactions [180]. While the addition of excipients such as arginine has been effective in mitigating aggregation, the destabilization of certain proteins remains a challenge. Alternatively, a flow-through mode, wherein the target antibodies flow through the resin and the impurities and are bound to the resin, can be used to purify therapeutic proteins, and due to this, it is also called a negative purification mode. Typically, the flow-through mode is carried out using anion exchange chromatography. This in turn shows great advantages over the positive purification system (bind and elute) with respect to overall time, buffer utilization and recovery rates [181].

This flow-through approach is particularly beneficial for recombinant proteins that are structurally or functionally sensitive to interactions with resins and has shown significant potential for continuous production processes. Yamada et al. reported that a complete flow-through purification strategy can simplify processing, reduce time, achieve low HCP levels (<100 ppm), and high purity for mAbs, offering a cost-effective alternative for antibody manufacturing. However, the reported yields were modest (42–47%) and required further optimization [182].

Lorek et al. demonstrated that flow-through mode with low partition coefficients (Kp) on anion exchange (AEX) and multimodal resins can achieve 99% protein recovery while reducing HCP levels by 23% (<100 ppm) for monovalent antibodies [183]. Similarly, Chen et al. employed a complete flow-through polishing strategy for scFv-containing bispecific antibodies, using mixed-mode hydrophobic interaction chromatography resins (Capto Butyl ImpRes) in combination with Capto adhere resins. This approach bypassed the high salt concentrations required in conventional bind-and-elute processes, resulting in high purity, HCP loads below 100 ppm (17–35-fold reduction post-rotein A elution), <1% high-molecular-weight species (HMW), and overall recoveries between 56 and 87% [89]. To minimize HCP contamination after purification, affinity tags can be attached to antibodies. Commonly used tags include Streptavidin, His, GST, arginine, Protein A and G, and FLAG tags. These tags can be attached to the N-terminus, C-terminus, or internal regions of the protein; however, they must be removed from the final product, typically using endopeptidases. While tag-assisted purification is widely employed for therapeutic proteins, it presents several challenges, including reduced protein quality, interference with host cell metabolism, aggregation, loss of functionality following proteolytic cleavage, and high costs associated with tag removal. Efficient tag removal is critical; as residual tags can provoke adverse immunogenic responses.

A promising alternative is a tag-free approach using self-cleaving protein tags mediated by inteins (intervening proteins). Inteins are self-splicing protein elements that excise themselves from precursor proteins, ligating the flanking N- and C-terminal exteins and some inteins are naturally split across two genes and are referred to as split inteins [184]. In this system, split inteins are co-expressed with the affinity tag and the target protein. Following affinity purification, the tag is removed through self-cleavage of the split intein, which can be induced by adjusting factors such as pH and temperature. The efficiency of cleavage is further enhanced by the addition of thiol groups [185].

Wu et al. demonstrated a single-step intein-based purification platform for two human antibody fragments containing disulfide bonds (EGF VH dAb and human light chain κ) expressed in *E. coli*. The constructs included a PelB leader sequence, a C-terminal intein (IntC), and a His tag. The IntC domain contains a chitin-binding domain (CBD), which becomes activated upon binding to chitin, triggering cleavage of the His tag and the intein while eluting the antibody in a tag-free form via pH-induced cleavage [186].

Beyond purification, split inteins can serve as a powerful high-throughput screening tool for evaluating large libraries of bispecific antibody formats generated during cloning. Hofmann and colleagues demonstrated this by high-throughput screening of a combinatorial pool of bsAb variants to identify constructs with minimal product-related impurities using Npu DnaE split inteins. In their approach, bsAb fragments were separately expressed in mammalian cells as His tag–IntN–Fab and His tag–IntC–Fc or one-armed (oa) SEED fragments. The fragments were then combined to reconstitute a functional intein, followed by affinity purification.

The split intein subsequently spliced itself, removing the His tags and intein sequences to yield the final bsAb product with comparable yield and purity to large-scale production [187]. While split inteins effectively preserve protein structure during processing, optimization of both product yield and cleavage efficiency remains essential to ensure scalable and robust manufacturing.

Table 6 shows the pros and cons of alternative methods used for purification and polishing steps.

## 6. Innovations in the Purification of Therapeutic Proteins

The precise removal of HCPs as well as aggregates formed during different stages of production of mAbs, bsAbs and other formats, remains a major unresolved challenge in downstream processing. Several novel strategies are currently under investigation aiming toward near-complete or maximized clearance of HCPs and structurally related impurities in mAb production, which could potentially be extended to bsAbs and related constructs. One of the examples is using a 3D-printed stationary phase chromatographic purification of IgG mAb in capture stages to tackle the costly adsorbents and buffer consumption. The columns consisted of a 30 µm microchannel structures and were functionalized with Protein A. For polishing steps, cation exchange ligand SO_3_ were utilized. The results after capture step were encouraging with >85% yields and 98% purity along with logarithmic reduction in HCPs (log reduction value > 2.5), and these results were observed. These 3D printed column designs could be reused for up to 20 cycles, thereby reducing the costs [203].

For effective HCP reduction, a high-performance counter-current membrane purification (HPCMP) was utilized by Mohammadzadehmarandi and group and showed a 100-fold reduction in HCPs in the final eluate and also achieved >95% of mAb yields within two days. The HPCMP, unlike other membranes, works on the principle of selective diffusion instead of relying on pressure and has a thin-walled hollow fiber structure with an added benefit of continuous downstream processing [196]. Further studies are required to understand the efficiency of HPCMP in purifying complex structures and in competing with traditional chromatography techniques.

A phase-separation-based technique, aqueous two-phase extraction (ATPE), has also shown promising results for mAb purification at laboratory scale [204]. IgG produced in CHO and PER.C6^®^ cell lines was purified using a continuous PEG/phosphate ATPE system. This approach achieved 80% and 100% yields, with 155-fold and 22-fold reductions in HCP/IgG ratios from CHO and PER.C6^®^ supernatants, respectively. In a separate study, the same authors also highlighted the cost-effectiveness of ATPE [205]. A key advantage of this method is the minimal interfacial stress on proteins, as it is based on liquid–liquid extraction, where biomolecules partition between two immiscible aqueous phases under defined conditions. The soluble component from the mixture will fall into the lower phase, and the proteins will be on the hydrophobic top phase. Furthermore, the proteins are segregated by adjusting the partition coefficient (K) factors such as hydrophobicity, ionic strength, molecular weight, affinity specificity and additions of salts [206]. In a much more recent study, APTE was utilized to purify an mAb of 150 kDa size, expressed in CHO cells. The author observed a >80% yield with 97% purity. However, for significant reduction in HCP, ion exchange chromatography was used in the polishing step to achieve HCP levels below 100 ppm [207]. Due to limited efforts in scale-up and industrial implementation, ATPE currently remains largely limited to laboratory-scale applications. Additional limitations include challenges in accurately predicting optimal partitioning conditions for different proteins and process systems.

Several researchers have also pointed out that precipitation-based capture using ZnCl2 and PEG target precipitation system. This was shown in a study for purification of therapeutic mAb with IgG1 structure. The precipitation was followed by solid–liquid extraction and the resultant yields of 79% and low HCP levels of 2 ppb and 99% purity were achieved by the team [208]. Rumanek and colleagues further applied a combination of multistage precipitation and solid–liquid extraction (SLE) to evaluate its effectiveness in purifying mAbs from clarified cell culture fluid [209]. PREC8-SLE consisted of a tubular flow precipitation reactor for continuous precipitation, wherein the cell culture fluid was added along with precipitating agents ZnCl2 and PEG. An optimal precipitation pH for separation of LMWI at pH 8 (PREC8) followed by SLE at pH 5 provided separation of HMWI. The overall purity of 99%, 78% yield and HCP reduction of 99.9%, i.e., from 300 µg/mL to 0.07 µg/ML was observed in a pilot study of clarified mAb with 3.0 mg/mL concentration [209]. This study showed two stage precipitation at pH 8 or PREC8-SLE potential to replace Protein A chromatography with equal to or better purity and HCP clearance and less time. This, however, needs to be studied in larger and complex antibody formats to establish it as a replacement for Protein A. Similar approach was utilized to analyze the capture and purification efficacy of three different commercially available mAbs viz., Adalimumab, Trastuzumab and Denosumab, wherein PEG was replaced with ZnCl2, as PEG makes the solution more viscous and thereby increases transmembrane pressure. Zinc has the ability to crosslink proteins to form a bigger aggregate, and using this principle 6 Mm of ZnCl2 was used in this study. The study showed 85% purity, <2% HMWI and lower HCPs (1246 ± 174 μg/mL to 160 ± 11 μg/mL) however similar yields of Adalimumab and Trastuzumab were observed as they are structurally similar (IgG1 type) but lower yields of Denosumab (IgG2 type) [210]. This indicates that further optimization is required, particularly for structurally diverse and more complex antibody formats, to fully establish the broader applicability of this precipitation-based capture strategy.

## 7. Limitations, Challenges and Future of Antibody-Based Therapy

Over several decades, antibody-based therapeutics have become a cornerstone of modern medicine due to their high specificity and ability to provide sustained therapeutic effects. However, their translation from concept to clinical application has revealed several biological and economic limitations. Biologically, the large molecular size of antibodies restricts their penetration across physiological barriers and limits their effectiveness against intracellular targets. In addition, despite high specificity, antibodies may elicit immunogenic responses [211], including the formation of anti-drug antibodies [212], and can exhibit relatively short half-lives compared to other therapeutic proteins. From an economic perspective, antibody production requires substantial capital investment due to complex upstream and downstream processing requirements [213]. These costs are further amplified by reduced long-term efficacy in areas such as oncology and by burdensome administration routes that can be time-consuming and inconvenient for patients.

As a result, there is a growing shift toward gene- and DNA-based therapeutic strategies. These approaches offer improved durability by targeting the genetic basis of disease and enabling potentially curative single-dose interventions. Their smaller molecular nature facilitates cellular uptake and access to intracellular machinery, enabling precise genome-level correction using technologies such as CRISPR. Moreover, their production relies on comparatively simplified manufacturing workflows, which can significantly reduce production and purification costs relative to antibody-based systems. Collectively, these next-generation modalities represent a promising and more efficient alternative, with the potential to substantially advance global healthcare outcomes.

## 8. Conclusions

Monoclonal antibodies (mAbs) and bispecific antibodies (bsAbs) have great potential for diagnosing and treating complex diseases, benefiting human health. However, producing and purifying these therapeutic proteins is still challenging, time-consuming, and expensive, creating a bottleneck in antibody-based therapy development. Most approved mAbs and bsAbs are produced in CHO cells, which inevitably introduce both process- and product-related impurities along with the target protein.

Chromatography remains the main method for downstream purification of antibodies. While it removes most impurities, trace amounts of host cell proteins (HCPs) can remain, which may reduce effectiveness or cause side effects in patients. To address this, it is important to both reduce impurity formation during production and fully remove remaining contaminants during purification. Specifically, several areas were highlighted for improving efficiency in antibody-based manufacturing: (1) selecting appropriate expression systems based on the antibody format and physicochemical properties to minimize yield loss (e.g., using CHO cells for complex glycosylated IgG antibodies or yeast systems for simpler antibody fragments); (2) developing high-producing cell lines through advanced genetic engineering approaches (e.g., CRISPR/Cas-mediated knockout of problematic host cell proteins such as proteases); (3) implementing robust host cell protein (HCP) detection and monitoring systems for accurate identification and characterization (e.g., combining ELISA with LC–MS-based proteomics for comprehensive HCP profiling); and (4) applying optimized purification strategies that ensure high yield and purity while preserving antibody structure, with consideration of transitioning toward continuous production workflows.

Apart from that, identifying rate-limiting steps in the current production strategy can provide room for further improvements. Table 7 shows rate-limiting obstacles faced in the production and purifications stages and possible improvements for an efficient production pipeline.

To conclude, creating a reliable and efficient production process for therapeutic proteins is still possible, especially with different antibody formats that can be optimized for specific needs. Improving production stability and purification methods is essential to support the development of new antibody-based drugs.

## Figures and Tables

**Table 1:** Steps involved in the downstream processes of antibody-based therapeutic production.

Process	Outcome
Harvesting	Clarify the cell culture by removing cells, debris, and insoluble particulates.
Capture chromatography	Isolate the target protein, remove bulk impurities, and concentrate the product.
Viral inactivation	Inactivate or remove potential viral contaminants.
Polishing	Reduce critical impurities to very low levels.
Ultrafiltration/diafiltration	Concentrate the target, exchange buffers, remove residual impurities, and finalize formulation.

**Table 2:** Equipment in antibody therapeutic manufacturing. The table summarizes main advantages and limitations.

	Process	Equipment	Function	Advantages	Limitations
**Upstream**	Media preparation	Mixing tanks/single-use mixers	Preparation of media and buffers under sterile conditions	Automated workflow ensures nutrient distribution and mixing	High maintenance requirements; pumps and sensors require frequent calibration
	Seed train	CO_2_ incubators /Wave bioreactors	Expansion of cell populations	Gentle rocking motion preserves cell viability; easy to handle for seed expansion	Limited oxygen transfer (kLa) compared to stirred tanks; scalability typically < 50 L
	Cultivation	Stirred-tank bioreactors (STR)	Maintains pH, dissolved oxygen (DO), and temperature	Highly scalable (>20,000 L); lower operating cost at high batch frequency; long equipment lifespan	High capital investment; time-consuming cleaning and sterilization between batches; risk of contamination
	Monitoring	Process analytical technology (PAT)	Inline monitoring (e.g., Raman spectroscopy, pH, DO)	High specificity and accuracy for process control	High capital cost; requires method standardization for different bsAb formats
**Harvest**	Clarification	Disk-stack centrifuge	Rapid separation of bulk cell mass from mAb-containing supernatant	Handles high cell densities (>50 million cells/mL); cost-effective for large batches (>2000 L) with low consumable usage	High rotational forces may cause shear stress to cells; major step for release of process-related impurities; expensive and requires intensive maintenance
		Depth Filters	Removal of fine cell debris, HCPs, and residual DNA	Gentle processing preserves protein integrity; effective removal of DNA and endotoxins	Clogging due to high debris load can cause fouling of systems
**Downstream**	Capture	Protein A chromatography column	Primary capture step using industry-standard affinity chromatography	Gold standard for mAb purification; high binding capacity (60–80 g/L)	High cost of Protein A resin
	Viral inactivation	Low pH hold tanks	Inactivation of enveloped viruses at pH 3.0–3.6	Simple process with strong regulatory acceptance	Requires long hold times; extended low-pH exposure may cause antibody aggregation
	Polishing	Anion/Cation exchange columns	Removal of aggregates, residual HCPs, and DNA	High load capacity of >100 g/L	Relatively slow; often a rate-limiting step
	Concentration	Ultrafiltration/ diafiltration (UF/DF)	Buffer exchange and concentration of proteins into final formulation	Precise and reliable; easily scalable by adding additional filter cassettes	High recirculation rates may cause shear stress and damage bsAbs; requires rigorous cleaning
	Sterilization	0.22 µm sterile filters	Final filtration prior to storage	Maintains product integrity; compatible with formulation buffers	Single-use nanofilters are expensive; high-concentration mAbs may cause filter clogging

**Table 3:** Common CHO-derived HCPs co-eluting with monoclonal antibodies.

Impact on Product	Host Cell Protein	References
Aggregation	78 kDa glucose-regulated protein	[31,32]
	Protein disulfide isomerase (PDI)	[30,33]
	Peptidyl-prolyl cis-trans isomerase A (PPIA)	[31,34]
Inflammatory response	Monocyte chemoattractant Protein-1 (MCP-1)	[31,35]
	Transforming growth factor-β1 (TGF-β1)	[33]
Degradative enzymes	Matrix metalloproteinase (MMP)	[31]
	Protein disulphide-isomerase A6 (PDIA-6)	[31,36]
Degradation of polysorbates	Carboxyesterase (CEB)	[37]
	Lipoprotein lipase (LPL)	[38]
	Lysosomal acid lipase (LAL)	[31,39]
	Lysosomal phospholipase A2 (LPLA2)	[34,40]
	Sialate o-acetylesterase (SIAE)	[31]
Fragmentation of drug	Cathepsins (B, L, Z)	[30,31]
	Cathepsin (D, E)	[30]
Immunogenic response	Annexin A5 (ANXA5)	[31,41]
	C-X-C motif chemokine 3 (CXCL3)	[31]
	Glutathione-S-transferase (GST)	[31]
	Clusterin (CLU)	[31,33]
	Peroxiredoxin (PRDX)	[40]
	Phospholipase B-like 2 (PLBL2)	[31,33]
	Procollagen-lysine 2-oxoglutarate 5-deoxygenase_1 (PLOD1)	[31]
	Protein S100	[36]
	Pyruvate kinase (PK)	[31]
Modification of drug	Alpha-enolase	[31,40]
	Carboxypeptidase D (Cpd)	[31]
	Serine protease (HTRA1)	[31]

**Table 4:** Summary of cell expression systems used for antibody-based therapeutic development.

Expression System	Cell Types	Advantages	Disadvantages	References
Mammalian	CHO	Human-like glycosylation and high production yields. Resistant to human viruses. Ideal for production of full IgG mAbs and bsAbs. Gold standard, ~80% of commercial therapeutic proteins are produced in CHO cells.	Can produce non-human glycosylation patterns, leading to potential immunogenic effects. Time-consuming with high production and purification costs. Genetic instability and incorrect assembly, especially in bsAbs.	[60,61]
	Murine (NSO)	Myeloma-derived cells with high secretion capacity. Lack glutamine synthetase (GS), enabling efficient positive selection systems.	Can produce galactose-α-1,3-galactose, which may trigger alpha-gal syndrome (AGS). Requires lipid-supplemented media.	[62,63]
	Human embryonic retinal cells	Provide folding patterns closely resembling native human proteins compared to yeast and murine systems Human-like glycosylation and PTMs, considered safer. Scalable up to ~500 mg/L.	No FDA-approved products yet; requires extensive validation and pilot studies for mAbs and complex bsAbs.	[64]
	HEK	Human glycosylation is beneficial for therapeutic antibodies. Suitable for complex bsAb structures. Fast production with high transfection efficiency. Considered one of the safest systems for antibody production.	Susceptible to human viruses (e.g., hepatitis B, minute virus of mice). Higher risk of aggregation in suspension culture. Increased N-glycan branching in Fc region, potentially reducing ADCC activity. Lower yields compared to CHO cells.	[65,66]
Bacterial	*E. coli*	High yield, fast production, and cost-effective compared to mammalian systems. Suitable for scFvs and nanobodies. No risk of human viral contamination. Several antibody therapeutics are in FDA phase III trials.	Lacks human glycosylation, leading to inclusion body formation. Endotoxin contamination is a major limitation. Cannot form correct disulfide bonds. Proteins are not secreted and accumulate as inclusion bodies in the cytoplasm. Requires complex refolding steps	[67,68,69,70]
Yeast	*P. pastoris.* *S. cerevisiae*	Proteins are secreted into extracellular medium, simplifying purification. Generally correct folding. Endotoxin-free system. Faster expression than mammalian systems. Suitable for BiTEs and Fab production.	Lacks human-like glycosylation and other PTMs. Produces high-mannose N-glycans, reducing serum half-life. Prone to misfolding and proteolytic degradation.	[71,72]
Cell free systems		Low cost and minimal infrastructure requirements. Eliminates contamination from genomic DNA. Rapid expression. Used in ADC production.	Lower yields compared to mammalian systems. Sensitive and fragile systems. High maintenance cost. Mainly used for screening studies; not standardized for large-scale production.	[73,74,75]

**Table 5:** Key parameters and applications of chromatographic methods.

**Affinity** **chromatography**	**Advantages:** Industry standard for mAbs; high purity (>95%); scalable up to 20,000 L; high dynamic binding capacity (65–80 mg/mL) for mAbs and IgG-based bsAbs; specialized ligands (Protein L, CaptureSelect) enable >98% purity with 85–90% yield.	[85,90,93,134,138,139,140,141,142,143,144,145]
	**Limitations:** Reduced binding for asymmetric bsAbs; harsh conditions (low pH, acids, ligand leaching) may affect stability; high resin cost (>$23,000); low-pH elution can induce aggregation; lower dynamic binding capacity (DBC) for aggregation-prone bsAbs reduces yield.	
	**Applicable for:** mAbs, antibody fragments (e.g., scFvs, half-antibodies), and bsAbs with Fc/Fab regions; homodimers, mono- and bivalent bsAbs, symmetric formats; engineered systems (KappaSelect, Fc-specific ligands)	
**Size-exclusion chromatography**	**Advantages:** Uses mild/native buffer conditions, minimizing structural instability; effective for removal of high- and low-molecular-weight impurities; relatively lower cost compared to affinity chromatography.	[2,97,100,146,147,148]
	**Limitations:** Lower selectivity compared to advanced affinity methods and hydrophobic interaction chromatography; slow flow rates and time-consuming; typically requires combination with other techniques for optimal yield; and highly concentrated samples.	
	**Applicable for:** mAbs and bsAbs prone to forming HMW and LMW impurities; final polishing steps, including removal of virus-sized particles (18–26 nm)	
**Hydrophobic interaction chromatography**	**Advantages:** Preserves tertiary structure and biological activity of mAbs and bsAbs; effective for aggregate removal; operates under non-denaturing conditions, supports higher sample loading compared to SEC; effective for hydrophobic HCP clearance.	[104,107,109,120,149,150,151,152]
	**Limitations:** Lower selectivity compared to affinity chromatography; high-salt elution may cause irreversible interactions with resins; potential conformational changes reported in bsAb; lower dynamic binding capacity (20–60 mg/mL) compared to affinity chromatography; typical yields range from 80 to 90%.	
	**Applicable for:** Removal of product-related isoforms and aggregates in bsAbs; purification of mispaired knob-into-hole bsAbs; separation of structural variants such as oxidized and misfolded mAbs.	
**Ion exchange chromatography**	**Advantages:** Highly scalable and widely used in large-scale manufacturing; low resin cost with improved HCP clearance; CEX resins provide high dynamic binding capacities (>90 mg/mL) for IgGs and scFvs; enables moderate to high purity for bsAb purification.	[112,113,152,153,154,155,156]
	**Limitations:** Requires significant isoelectric point differences for bsAb separation; sensitive to pH and conductivity fluctuations; method optimization is time-consuming; low efficiency for symmetric bsAbs or balanced charge-distributed formats.	
	**Applicable for:** bsAbs with charge variants, including homodimers, knob-into-hole (KiH) constructs, and aggregated species; final polishing steps in purification.	
**Mixed-mode** **chromatography**	**Advantages:** Highly effective in HCP clearance (up to 99% for mAbs); calcium hydroxyapatite enables high purity separation of HMW, LMW, and problematic impurities (e.g., PS-80) in bsAbs; high dynamic binding capacity for mAbs (up to 97 mg/mL) and >100 mg/mL for asymmetric bsAbs; lower resin cost.	[126,130,145,157,158]
	**Limitations:** Requires complex optimization due to multiple interaction modes, making the process time-consuming and moderately scalable; hydrophobic interactions in MMC may promote aggregation.	
	**Applicable for:** mAbs across formats for both capture and polishing; asymmetric IgG-based bsAbs, including separation of half-antibodies, hole–hole homodimers, and light chain mispairs.	

**Table 6:** Summary of alternate approaches for purification and polishing steps.

**Multicolumn (MCC) vs. Batch**	**Advantages:** 40–80% higher resin utilization compared to batch processes. Reduced buffer consumption. Lower recurring costs compared to batch processes. Approximately 30% increase in productivity. Short residence times in buffers, which can help reduce denaturation of bsAb structures. Continuous processing enables increased productivity. Cost reduction of up to 35% has been estimated when switching from batch to continuous processing.	[121,138,188,189]
	**Limitations:** It requires high capital investment, carries an increased risk of contamination, cannot process sample concentrations above 20 g/L, and demands intensive maintenance.	
**Multicolumn counter current solvent gradient purification (MCSGP)**	**Advantages:** High yield (up to 90%) and purity (~95%) can be achieved for mAbs and bsAbs due to internal recycling. Buffer consumption can be reduced by up to 90%. Reduced resin requirements and smaller column sizes with lower stationary phase volumes enable scalable production. Less labor-intensive workflow, with continuous processing enabling improved process monitoring and time efficiency. Gradient-based elution reduces the risk of contamination.	[165,166,190,191,192]
	**Limitations:** High initial investment, skilled personnel, and well-established process planning are required. Continuous workflows offer limited flexibility and must be carefully designed in advance. This approach is most suitable for intermediate and polishing steps.	
**Periodic counter-current chromatography**	**Advantages:** Well-suited for capture steps using Protein A columns, achieving up to 98% mAb purity. BsAbs can be obtained with yields exceeding 83% and purity levels of up to 98%. Exhibits higher resin utilization compared to MCSGP. Employs step elution, making it more suitable for large-scale purification. Simpler in operation compared to MCSGP.	[168,178,193,194]
	**Limitations:** Reduction in HCPs is lower compared to traditional batch chromatography. Further optimization is required to achieve higher yields and accommodate larger feed volumes, e.g., 20–30 L/day.	
**High-performance tangential** **flow filtration (HP-TFF)**	**Advantages:** Separation is based on protein size and charge, making it suitable for single-step purification models. mAb yields of ≥95% have been reported. Absence of recirculation reduces the risk of contamination and shear stress. Membranes can be reused for up to seven cycles.	[177,195,196,197]
	**Limitations:** Large-scale implementation requires extensive membrane surface area, buffers, and processing time. Membrane fouling remains a key issue that needs to be addressed. Single-pass TFF systems have demonstrated low HCP clearance efficiency. Scale-up is challenging due to the limited availability of suitable membranes. Scaling up the purification process is challenging due to the limited availability of suitable membranes.	
**Charged ultrafiltration (UF) membranes**	**Advantages:** Relatively new in mAb purification applications but has demonstrated purity levels of up to 96%. Efficient in separating antibodies with similar molecular weights. Suitable for concentration and final polishing steps.	[198,199]
	**Limitations:** Not suitable for capture stages, as impurities may be co-transported. Limitations include membrane fouling, concentration polarization, and reduced process efficiency.	
**Split inteins**	**Advantages:** Has shown promising results in addressing chain mispairing. Maintains the structural integrity of complex molecules such as bsAbs after splicing. Employs mild reducing agents that do not damage, alter the final product. Suitable for high-throughput screening of bsAbs.	[187,200,201,202]
	**Limitations:** Sensitive to extein sequences; therefore, detailed knowledge of the sequence is essential. Industrial-scale manufacturing potential has not yet been fully explored. The two-step process required to produce separate precursor fragments leads to increased production costs. Premature cleavage can result in the formation of process-related impurities.	

**Table 7:** Key rate-limiting steps in therapeutic antibody production and strategies for their mitigation.

Step	Limitations	Mitigation
**Upstream expression**	Accumulation of metabolic by-products	Timely monitoring of by-product accumulation; media optimization by adding supplements such as L-glutamine and trace minerals; control of process parameters such as humidity and agitation speed [214].
	High media requirements for high-producing cell lines.	Media optimization and cost-efficient feed strategies.
	Limited oxygen transfer rates in large bioreactors, leading to non-uniform distribution.	Improved bioreactor design and enhanced aeration strategies.
**Harvesting**	Shear stress caused by mixing tanks leading to increased release of HCPs.	Identification of shear stress limits for specific cell lines and use of alternative technologies such as acoustic wave separation [215].
	High-producing cells generate dense slurries that may lyse during centrifugation, releasing HCPs.	Optimization of operating conditions and use of microfiltration systems to reduce cell damage [216].
	Thick slurries can clog reactors, pipes, and filters.	Regular maintenance and cleaning of equipment after each cycle to ensure efficient performance.
**Downstream purification**	High production yields require large Protein A columns.	Use of multimodal and continuous processing to improve binding efficiency and reduce column size requirements.
	High buffer consumption.	Buffer recycling and process intensification strategies.
	Membrane fouling	Regular cleaning and replacement of membranes, although associated with high cost.
	Limited sensitivity in HCP detection between process steps.	Improvement of analytical detection kits and monitoring methods.
**Polishing step**	Aggregates of mAbs and bsAbs may remain bound after polishing.	Use of mixed-mode chromatography or combination of multiple chromatographic techniques.
	Viral clearance challenges due to small virus size relative to antibodies, allowing possible passage through polishing steps.	Although polishing limits are reached, Planova 15N and 20N filters have demonstrated up to 4-log reduction in parvovirus [217].

## Data Availability

This study did not generate or analyze any new data. Therefore, data sharing is not applicable.

## Abbreviations

The following abbreviations are used in this manuscript:

AEX	Anion Exchange Chromatography
bsAbs	Bispecific Antibodies
CEX	Cation Exchange Chromatography
HC	Heavy Chain
CHO	Chinese Hamster Ovary
CHT	Ceramic Hydroxyapatite
DBC	Dynamic Binding Capacity
DHFR	Dihydrofolate Reductase
ELISA	Enzyme-Linked Immunosorbent Assay
EpCAM	Epithelial Cell Adhesion Molecule
Fab	Fragment Antigen-Binding
Fc	Fragment Crystallizable
HCP/HCC	Host Cell Proteins/Contaminants
HEK	Human Embryonic Kidney
HIC	Hydrophobic Interaction Chromatography
HP-TFF	High-Performance Tangential Flow Filtration
IEX	Ion Exchange Chromatography
KiH	Knob-Into-Hole
LC-MS	Liquid Chromatography–Mass Spectrometry
mAbs	Monoclonal Antibodies
MCC	Multicolumn Chromatography
MCSGP	Multicolumn Counter-Current Solvent Gradient Purification
MMC	Mixed-Mode Chromatography
mS/cm	MilliSiemens per Centimeter
PCC	Periodic Counter-Current Chromatography
PTM	Post-Translational Modifications
scFv	Single-Chain Variable Fragment
SEC	Size-Exclusion Chromatography
SMB	Simulated Moving Bed
SP-TFF	Single-Pass Tangential Flow Filtration
TFF	Tangential Flow Filtration
VH	Variable Heavy Chain
VL	Variable Light Chain
WPC	Weak Partitioning Chromatography
